# Supplementary material for: Do Teachers Question the Reality of Pain in Their Students? A Survey Using the Concept of Pain Inventory-Proxy (COPI-Proxy)
Source: Children (Basel). 2023 Feb 13;10(2):370. doi: 10.3390/children10020370 (PMC9955080; doi:10.3390/children10020370)
Supplement: Supplementary file 1 [file children-10-00370-s001.zip › File S1.pdf]

Supplemental digital content (SDC) 1:

Concept of Pain Inventory-Proxy (COPI-Proxy)

Fechner, R., Noel, M., Verhagen, A., Turbitt, E., Pate, J.W. (2022)

<u>Description:</u> The Concept of Pain Inventory-Proxy (COPI-Proxy) is designed to assess the concept of another person's pain. This version (with attached vignette) has been adapted for teachers to assess their concept of their student's pain.

<u>Scoring:</u> The following scale should be used for all 14 items. No items should be reverse scored.

0 = Strongly Disagree
1 = Disagree
2 = Unsure
3 = Agree
4 = Strongly Agree

Higher COPI-Proxy scores reflect greater alignment with contemporary pain science (Total scores can range from 0-56).

Please contact Rebecca Fechner at email: rebecca.fechner@student.uts.edu.au if you would like further information or would like to contribute to a shared database being built.

Concept of Pain Inventory-Proxy (COPI-Proxy)

Rebecca Fechner, Melanie Noel, Arianne Verhagen, Erin Turbitt, Joshua W Pate.

<u>Please read the following scenario before completing the questionnaire:</u>
Imagine you have a student named Sarah in your class. She is 12 years old and is complaining of stomach pain after coming in from the midday break. She has reported stomach pain before, but in the past you have been able to distract her so she has been able to continue with the day. Today, Sarah is crying and wants to go home because of her pain.

Instructions: These sentences are about what you think pain is for Sarah, why Sarah feels pain, and how Sarah feels pain. Please read each sentence carefully. Indicate how much you agree or disagree with each sentence.

Items	Strongly disagree	Disagree	Unsure	Agree	Strongly agree
1. Feeling stressed can make Sarah feel more pain	○	○	○	○	○
2. Feeling sad can make Sarah feel more pain	○	○	○	○	○
3. Being distracted can make sarah feel less pain	○	○	○	○	○
4. Doing something Sarah enjoys could make her feel less pain	○	○	○	○	○
5. Sarah's pain is a warning that her body needs to be protected	○	○	○	○	○
6. If Sarah felt pain for a long time, her brain can become more sensitive to warning messages	○	○	○	○	○
7. Sarah can feel a lot of pain, even when an injury is small	○	○	○	○	○
8. Learning about pain can help sarah feel less pain	○	○	○	○	○
9. Sarah can have an injury and feel no pain	○	○	○	○	○
10. Sarah's brain can make her pain better or worse	○	○	○	○	○
11. Sarah could feel a little bit of pain even when an injury is big	○	○	○	○	○
12. Sarah could be feeling pain even after an injury heals	○	○	○	○	○
13. Pain usually feels better if Sarah moves her body a little bit more each day	○	○	○	○	○
14. Sarah's brain processes lots of details before she feels pain	○	○	○	○	○
